# Comparing the Utility of Mitochondrial and Nuclear DNA to Adjust for Genetic Ancestry in Association Studies

**DOI:** 10.3390/cells8040306

**Published:** 2019-04-03

**Authors:** Brendan Miller, Thalida E. Arpawong, Henry Jiao, Su-Jeong Kim, Kelvin Yen, Hemal H. Mehta, Junxiang Wan, John C. Carpten, Pinchas Cohen

**Affiliations:** 1 Leonard Davis School of Gerontology, University of Southern California, Los Angeles, CA 90089, USA; brendajm@usc.edu (B.M.); arpawong@usc.edu (T.E.A.); henrygji@usc.edu (H.J.); sujkim@usc.edu (S.-J.K.); kelviny@usc.edu (K.Y.); hemalmeh@usc.edu (H.H.M.); junxianw@usc.edu (J.W.); 2 Department of Translational Genomics and Institute for Translational Genomics, Keck School of Medicine of the University of Southern California, Los Angeles, CA 90033, USA; carpten@usc.edu

**Keywords:** genome-wide association study, DNA, mitochondrial, polymorphism, single nucleotide, principal component analysis, genetics, population

## Abstract

Mitochondrial genome-wide association studies identify mitochondrial single nucleotide polymorphisms (mtSNPs) that associate with disease or disease-related phenotypes. Most mitochondrial and nuclear genome-wide association studies adjust for genetic ancestry by including principal components derived from nuclear DNA, but not from mitochondrial DNA, as covariates in statistical regression analyses. Furthermore, there is no standard when controlling for genetic ancestry during mitochondrial and nuclear genetic interaction association scans, especially across ethnicities with substantial mitochondrial genetic heterogeneity. The purpose of this study is to (1) compare the degree of ethnic variation captured by principal components calculated from microarray-defined nuclear and mitochondrial DNA and (2) assess the utility of mitochondrial principal components for association studies. Analytic techniques used in this study include a principal component analysis for genetic ancestry, decision-tree classification for self-reported ethnicity, and linear regression for association tests. Data from the Health and Retirement Study, which includes self-reported White, Black, and Hispanic Americans, was used for all analyses. We report that (1) mitochondrial principal component analysis (PCA) captures ethnic variation to a similar or slightly greater degree than nuclear PCA in Blacks and Hispanics, (2) nuclear and mitochondrial DNA classify self-reported ethnicity to a high degree but with a similar level of error, and 3) mitochondrial principal components can be used as covariates to adjust for population stratification in association studies with complex traits, as demonstrated by our analysis of height—a phenotype with a high heritability. Overall, genetic association studies might reveal true and robust mtSNP associations when including mitochondrial principal components as regression covariates.

## 1. Introduction

Mitochondrial genome-wide association studies (MiWAS) are used to identify mitochondrial single nucleotide polymorphisms (mtSNPs) that associate with disease or disease-related phenotypes. A human mitochondrion has several copies of a condensed circular genome that encodes 13 large proteins, 22 tRNAs, 2 rRNAs, and many peptides (e.g., humanin, MOTS-c, and SHLPs) [1,2,3,4,5,6]. Genetic variation in these genes could alter mitochondria function and increase risk for certain diseases in specific ethnicities, as mitochondria DNA (mtDNA) reflects historical human migration patterns. Mitochondria genetic variation began to rapidly disseminate across the globe approximately 100,000 years ago. For instance, the estimated age of American-defined haplogroups of A, B, C, and D are just 25,000–50,000 years old, whereas the African-originating haplogroups of L2 and L3 are approximately 70,000-80,000 years old [7]. Given the rapid pace of mitochondrial genetic variation, it is plausible that mtSNPs help explain health disparities in ethnicities and are therapeutic targets for disease care and prevention. 

Several mtSNPs have been associated with metabolic disease and neurodegenerative disease risk. For example, Kraja et al. reported that two mtSNPs significantly associated with metabolic outcomes in a MiWAS that included ~170,000 individuals from 45 cohorts [8]. Additionally, our group identified a mtSNP in the humanin-coding region that associates with cognitive impairment and lower circulating humanin levels predominantly in Black Americans. We also showed that administering humanin in vivo improved cognition and attenuated neuroinflammation in aging mice [9]. More associations between mtSNPs and Parkinson’s disease, Alzheimer’s disease, diabetes, and other chronic diseases have also been noted with and without ample validation [10,11,12,13]. Hence, investing in MiWAS and related association techniques could be an invaluable tool to identify ethnic-specific mtSNPs that increase risk for disease. 

However, mitochondrial and nuclear genetic association studies are prone to confounding associations because of population substructures embedded within the studied samples if drawn from multi-ethnic or admixed groups [14]. In order to statistically adjust for differences in population substructure, principal components are calculated as eigenvalues from nuclear DNA (nucDNA) principal component analysis (PCA) and included in regression analyses as covariates. PCA is a statistical method whereby the number of variables that characterize variation in the data (e.g., number of SNPs in ancestral subgroups) is reduced to a smaller number of principal components that similarly represent the variation. For MiWAS, groups have statistically adjusted for genetic ancestry in the regression models by using varying numbers of these principal components calculated from either nucDNA or mtDNA [15,16,17,18]. Furthermore, there is no standard method when controlling for genetic ancestry during mitochondrial and nuclear genetic interaction scans, especially across ethnicities with heterogeneous mitochondrial genetic ancestry. A recent analysis showed that mtDNA principal components recapitulated mitochondrial haplogroups and outperformed haplogroup and nucDNA PCA when adjusting for genetic ancestry in simulated phenotype/mtSNP analyses [19]. These observations provide rationale to evaluate how well mtPCA recapitulates self-reported ethnicity compared to nucPCA, which has yet to be done in such a large, nationally representative, longitudinal, and multi-ethnic cohort. Comparing the utility of nucPCA and mtPCA will inform researchers who use PCA to adjust for genetic ancestry in genetic association studies. 

The purpose of analyses presented here is three-fold: Use a large cohort with a high-level of admixture to (1) assess whether White, Black, and Hispanic individuals can be grouped into ancestral clusters using principal components derived from array-based mtSNPs, (2) compare nucDNA and mtDNA representation of ethnic variation, and (3) examine effects of principal components derived from mtPCA on height—a phenotype with a high heritability—in White, Black, and Hispanic Americans [20].

## 2. Materials and Methods

### 2.1. Data

Data from the Health and Retirement Study (HRS), an on-going nationally representative study of United States adults over 50 years-old, was used for all analyses. The goal of HRS is to track changes in aging-related outcomes over time. HRS has genotyped nearly 16,000 individuals using either the Illumina HumanOmni2.5-4v1 and HumanOmni2.5-8v1 arrays for samples collected in 2006, 2008, and 2010. Genotyping was performed by the NIH Center for Inherited Disease Research (CIDR, X01HG005770-01) with standard quality control procedures implemented by the University of Washington Genetic Coordinating Center [21,22]. The total number of SNPs retained were those that overlapped across arrays and passed quality control standards, yielding 2,315,518 nucSNPs and 90 mtSNPs. Frequency of mtSNPs is listed in Table S1. 

### 2.2. Principal Component Analysis on mtSNPs and nucSNPs

Principal component analysis was conducted separately using mtSNPs and nucSNPs in ethnic-stratified and ethnic-combined analysis (i.e., PCA for combined ethnicities and PCA exclusively for White, Black, and Hispanic individuals). For nucSNPs (coded 0, 1, 2), the PLINK 2.0 pca command was used to extract principal components. In this process, nucSNPs are used to calculate eigenvectors using a variance-standardized genetic relationship matrix between individuals, which is similar to that implemented in EIGENSTRAT software [23]. No clumping by linkage disequilibrium was conducted prior to nucPCA because there is no way to ensure the compatibility in pruning processes across both nucSNPs and mtSNPs. For binary coded mtSNPs (coded 0, 1), the prcomp function in R was used to generate principal components. The prcomp function uses singular value-decomposition of the data matrix to provide eigenvectors that are the closest approximation of the matrix using a minimum number of values [24]. A total of 2,315,518 nucSNPs and 90 mtSNPs were used for the analyses. Visualizations of PCA plots were generated by standardizing components to a mean of zero with a standard deviation of 1 and by using the scatterplot3D and ggplot2 package in R (version 3.5.1, R Foundation, Vienna, Austria, 2018). 

### 2.3. Machine Learning Decision-Tree Classification of Self-Reported Ethnicity Using nucPCA and mtPCA

The caret R package, which contains a group of functions to create predictive models, was used to generate a cross-validated (kfold = 10; repeat = 5) decision-tree training algorithm on 30 percent of the data (*n* = 4584) by using 20 nuclear and/or 20 mitochondrial principal components. The optimal model was selected using the largest accuracy value derived from the train function (rpart method and a tune length of 10) and subsequently predicted self-reported ethnicity on the remaining 70 percent of the data using the predict function. Plots were generated using the prp function of the rpart.plot R package. 

### 2.4. Effects of Mitochondrial Principal Components on Height

Effects of mtSNP principal components on height was estimated by constructing multivariable linear regression models separately for each ethnic group (White, Black, and Hispanic Americans) and in a combined ethnicity model using the lm function in R. The dependent variable was height (in centimeters) and the predictors included a total of 20 principal components, biological sex, and centered age. The purpose of these analyses was to (1) understand whether reducing mitochondrial genetic variation with principal components could explain the variation in height within and across ethnic groups and (2) serve as proof-of-concept for using mtSNPs to characterize genetic ancestry in association studies. 

## 3. Results

### 3.1. HRS Sample Characteristics

The race/ethnic makeup of the study sample is presented in Table 1. Self-reported Whites made up the majority of the sample (70.2%), followed by Blacks (15.9%), Hispanics (11.2%), and Other (2.7%). 

### 3.2. Mitochondrial and Nuclear Principal Component Analysis

#### 3.2.1. Inter-Ethnic Analysis

**Figure 1:**
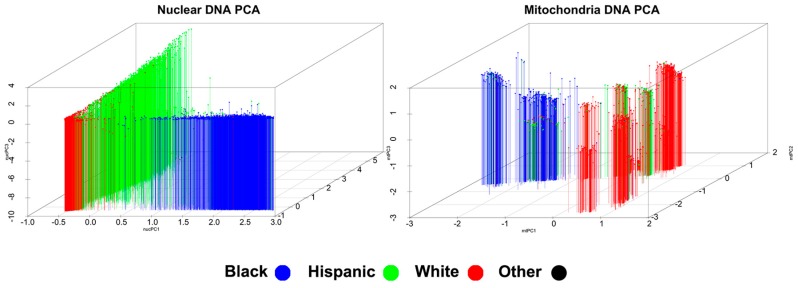
Multi-ethnic principal component analysis on nuclear and mitochondrial single nucleotide polyrmophisms. Each data point represents one individual. Colors are coded by self-reported ethnicity.

Nuclear PCA revealed that the first three principal components captured the highest amount of variance and, when these three were plotted, reflected an expected pattern of ancestry. As shown in Figure 1 in the left-hand plot, self-reported Whites, Blacks, and Hispanics clustered in the lower left corner, lower right, and along the left plane, respectively, with those in the Other group being dispersed across clusters. Variance explained by the first five nuclear principal components totaled 94.0 percent.

Mitochondrial PCA revealed an expected pattern of ancestry when plotting the first three principal components. Also shown in Figure 1, in the right-hand plot, self-reported Whites were grouped in separate clusters on the top right and lower right; Blacks grouped into clusters on the left midline; Hispanics grouped adjacent to the top right cluster of Whites; and those in the Other group were again dispersed across clusters. Variance explained by the first five mitochondrial principal components totaled 58.7 percent. 

#### 3.2.2. Intra-Ethnic Analysis

**Figure 2:**
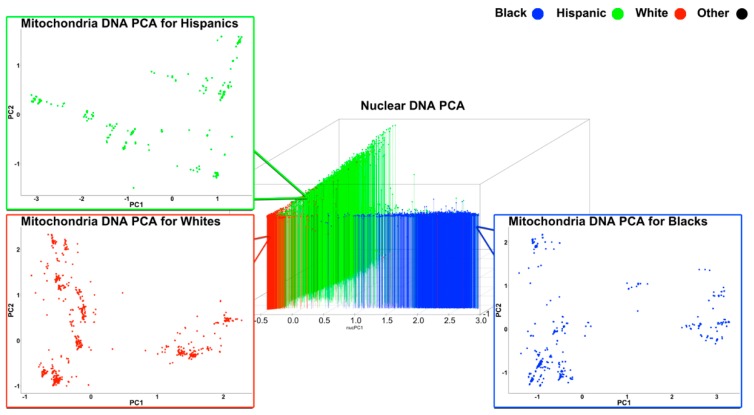
Intra-ethnic principal component analysis on mitochondrial SNPs. Red indicates Whites; Blue indicates Blacks; and Green indicates Hispanics.

The analysis of intra-ethnic mtPCA revealed population substructures. As seen in Figure 2, several sparse substructures were identified by conducting mtPCA within just Whites, Blacks, and Hispanics (Figure 2). These data suggest that mtPCA captures genetic variation even within White, Black, and Hispanic subgroups, which is informative for researchers attempting to examine the effect of mtSNPs during mitochondrial gene association studies and nuclear/mitochondrial interaction genetic association studies.

**Figure 3:**
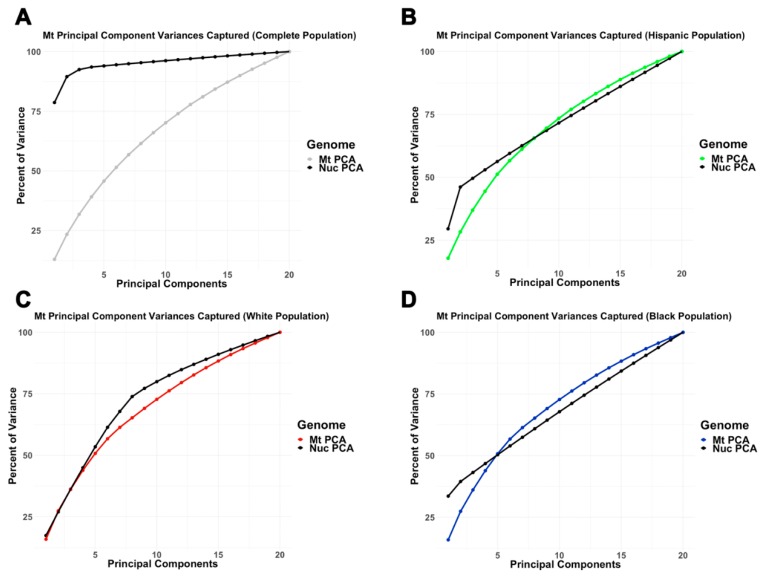
Comparing the amount of variance captured by nuclear and mitochondrial principal components (**A**) across all ethnicities, (**B**) among Hispanic, (**C**) among White, and (**D**) among Black samples.

The amount of variation captured by nucPCA and mtPCA was examined by self-reported ethnicity. While inter-ethnic nucPCA captured much more variance in fewer components than mtPCA, intra-ethnic mtPCA captured similar to slightly more variance compared to nucPCA (Figure 3). In particular, slightly greater variation was captured within the first 10 mitochondrial principal components for Hispanics (nucPC1:10 = 71.6%; mtPC1:10 = 74.8%) and Blacks (nucPC1:10 = 67.8%; mtPC1:10 = 72.7%), but not in Whites (nucPC1:10 = 80.0%; mtPC1:10 = 71.1%). 

### 3.3. Using Nuclear and Mitochondrial Principal Components for Ethnic Subgroup Classification

**Figure 4:**
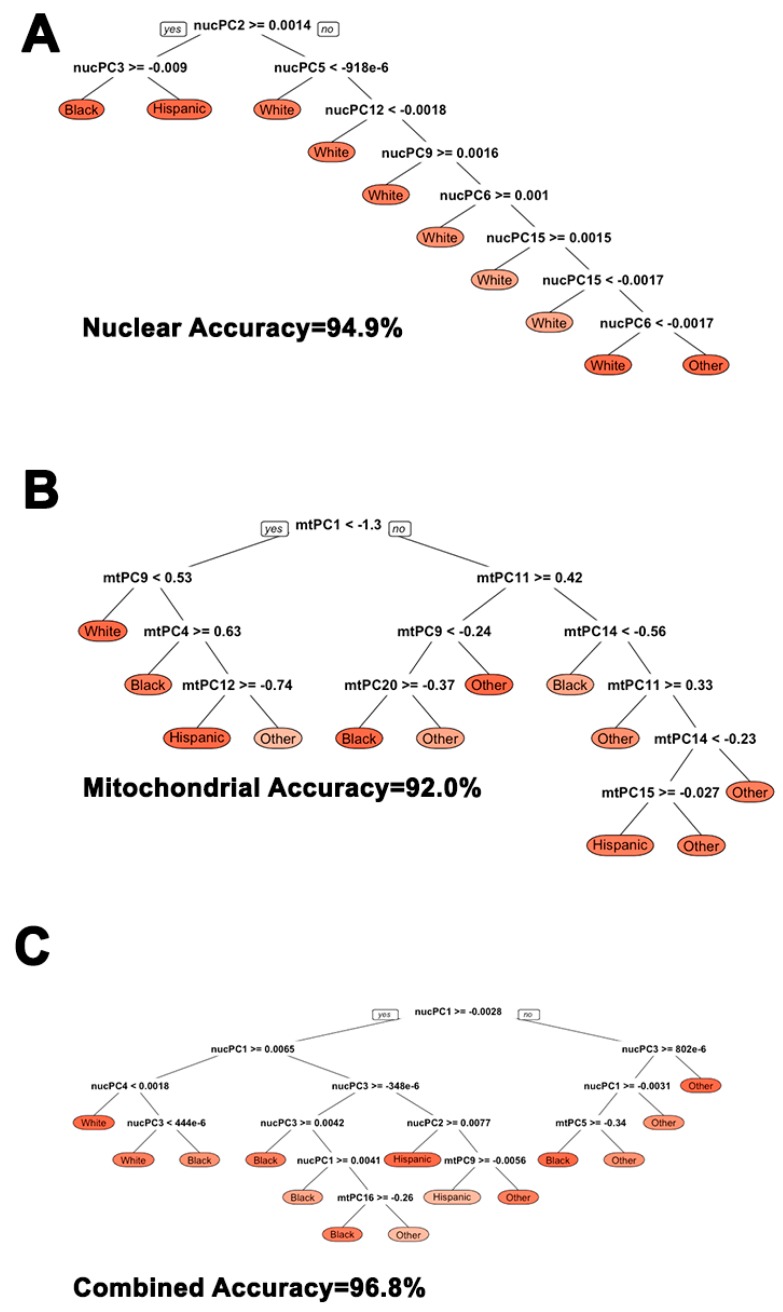
Comparison of nuclear, mitochondrial, and combined nuclear/mitochondrial PCA for statistically classifying individuals into broader ethnic sub-groups. (**A**) nucPCA shows a statistical classification accuracy rate of 94.9%; (**B**) mtPCA shows a statistical classification accuracy rate of 92.0%; (**C**) Combined nuclear and mtPCA shows a statistical classification accuracy rate of 96.8%.

We assessed how well nuclear and mitochondrial principal components classified broader ethnic sub-groups when defined by self-report. Using optimal decision tree algorithms derived from a 30% training sample and then separately implemented on the remaining 70% of the data, we found that nuclear and mitochondrial principal components comparably classified individuals into ethnic sub-groups to a high degree: at a 94.9 percent and 92.0 percent rate, respectively. Combining both nuclear and mitochondrial principal components increased statistical classification accuracy into self-reported ethnic subgroups to 96.8 percent. This analysis is notable because the nuclear and mitochondrial misclassification error suggests controlling for genetic ancestry within self-reported ethnic analyses or assigning individuals into genetically homogenous groups for analyses is necessary. Cutoffs and nodes are illustrated in Figure 4.

### 3.4. Effect of Mitochondrial Principal Components on Height

**Figure 5:**
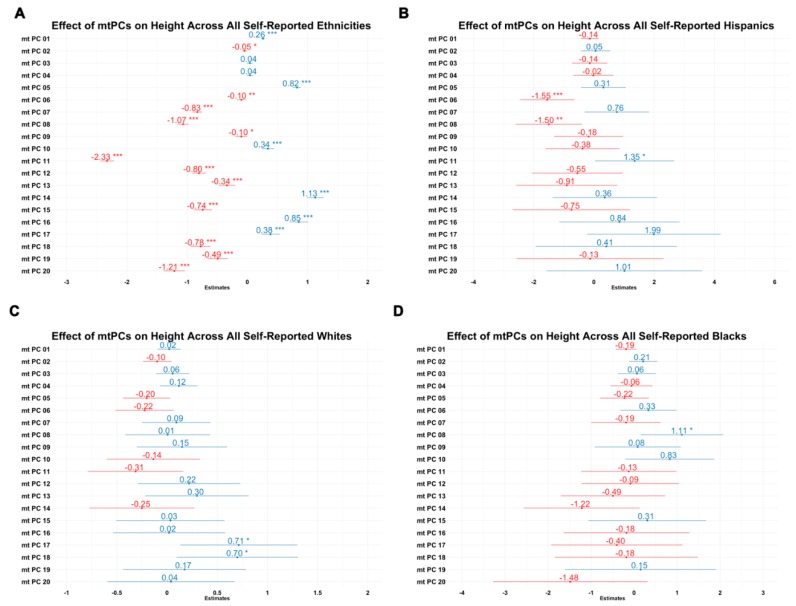
Effects of 20 mitochondrial principal components on height (centimeters) in inter-ethnic and intra-ethnic samples. (**A**) Combined analysis; (**B**) Hispanic analysis; (**C**) White analysis; (**D**) Black analysis. X axes are the coefficient estimate for mitochondrial principal component number (Y axes). * *p* < 0.05; ** *p* < 0.01; *** *p* < 0.001.

In order to evaluate the utility of mitochondrial principal components as covariates to adjust for population stratification in association studies, we tested its association with height (Figure 5), which is highly heritable and strongly linked to ancestry. In these analyses, 18 of 20 mitochondrial principal components derived from complete ethnic PCA significantly predicted height. This supports the strong association between height and ancestry markers. In intra-ethnic analysis, in which we used mitochondrial principal components derived from separately conducted sub-ethnic PCA, three components were significant among Hispanics, one component was significant among Blacks, and two components were significant among Whites. These analyses suggest that mitochondrial genetic dimension reduction strategies could be useful for identifying mtSNPs that associate with phenotypes in mitochondrial-specific analyses such as MiWAS. 

## 4. Discussion

Our analyses demonstrated the utility of mtPCA for mitochondrial and nuclear genetic association studies. First, we showed genetically admixed substructures from mtDNA in all ethnicities in HRS. Second, we illustrated that the amount of variance captured by mitochondrial principal components in Hispanics and Blacks is similar to slightly greater than that captured by nuclear principal components, whereas nuclear principal components captured substantially more variance in combined ethnic analysis and in Whites. Third, using mitochondrial and nuclear principal components to train a decision tree for self-reported ethnicity classification showed high statistical accuracy yet similar misclassification error between mitochondrial and nuclear analyses. This misclassification rate suggests that conducting MiWAS by ethnic-specific stratification without adjusting for genetic ancestry might not be a sufficient way to control for genetic admixture. Hence, we showed that factoring in principal components during stratified analysis can provide an analytic approach to further address the more complex admixture. Our analysis shows that mitochondrial principal components associated with a high heritability phenotype, height, when evaluated across ethnicities and intra-ethnically. 

One novel aspect of our analyses is that mtSNPS derived from an array capture within ethnic variation, which could be critical when designing analytic strategies to minimize confounding due to admixture. In the absence of nuclear DNA data during mitochondrial gene association studies (e.g., targeted whole mitochondria DNA sequencing), controlling for genetic ancestry using mitochondrial principal components could reduce type one error and provides a solution for analyses lacking nuclear DNA. 

As nationally representative cohorts continue to grow larger, it is likely that research groups will attempt to identify the effects of mtSNPs on a variety of phenotypes. Based on previous publications, groups might design their analytic strategies by assigning terms to mitochondrial haplogroups or single mtSNPs while controlling for genetic ancestry. The former is limited by reference group classification and the latter is limited by no standard method to control for genetic ancestry. Notably, Biffi et al. examined mtSNPs derived from commercially based arrays—similar to that used by HRS— and showed that mitochondrial haplogroup analysis was inferior to mtPCA for discovery of true associations and nucPCA had little effect on mitochondrial association testing [19]. Since prior groups who conducted MiWAS have controlled for genetic ancestry using nucPCA, it is possible that the loss of degrees of freedom from the addition of unnecessary nuclear principal components suppressed mitochondrial genetic associations and/or limited estimates of mitochondrial genotypes. 

Future mitochondrial gene studies might reveal true and robust mtSNP associations by controlling for mitochondrial genetic principal components. Moreover, it is plausible that there is significant interaction between nuclear and mitochondrial SNPs, and that controlling for mitochondrial genetic ancestry to identify such nuclear and mitochondrial genetic associations might be an important consideration when defining the analytic strategy. However, subpopulation genetic architecture will vary from cohort-to-cohort due to SNP-based array and sample variation. Therefore, before conducting genetic association studies, comparing nuclear and mitochondrial genetic substructures by ethnicity could guide analytic plans. 

## Figures and Tables

**Table 1:** Health and Retirement Study Sample Characteristics.

Race/Ethnicity	N
White	10,963
Black	2,488
Hispanic	1,753
Other	414

## Supplementary Materials

The following are available online at https://www.mdpi.com/2073-4409/8/4/306/s1, Table S1: mtSNP Frequencies in HRS.
