# Supplementary material for: Comparing the Utility of Mitochondrial and Nuclear DNA to Adjust for Genetic Ancestry in Association Studies
Source: Cells. 2019 Apr 3;8(4):306. doi: 10.3390/cells8040306 (PMC6523867; doi:10.3390/cells8040306)
Supplement: Supplementary file 1 [file cells-08-00306-s001.pdf]

Supplement Data

A Comparison of Mitochondrial and Nuclear DNA Utility for Controlling Genetic Ancestry in Association Studies

Brendan Miller [1], Thalida E. Arpawong[1], Henry Jiao [1], Su-Jeong Kim[1], Kelvin Yen[1], Hemal M. Mehta[1], Junxiang Wan[1], John C. Carpten[2], Pinchas Cohen[1*]

[1] Leonard Davis School of Gerontology, University of Southern California, Los Angeles, CA
[2] Department of Translational Genomics and Institute for Translational Genomics, Keck School of Medicine of the University of Southern California, Los Angeles, California
* Correspondence: hassy@usc.edu

Table S1: mtSNP Frequencies in HRS

mtSNP	Frequency
MitoT217C	1.14%
MitoG247A	4.54%
MitoC295T	7.74%
MitoC458T	4.08%
MitoT479C	1.93%
MitoT491C	11.40%
MitoG752A	2.04%
MitoC1050T	0.87%
MitoT1191C	4.47%
MitoG1440A	4.14%
MitoG1721A	4.57%
MitoT2160C	0.97%
MitoG2708A	30.88%
MitoG3012A	18.90%
MitoA3349G	0.33%
MitoT3395C	0.87%
MitoA3721G	1.04%
MitoG3916A	1.76%
MitoG3919A	1.52%
MitoC3971T	0.25%
MitoC3993T	1.48%
MitoA4825G	3.95%

MitoC4884T	0.87%
MitoA4918G	7.78%
MitoT4978C	2.34%
MitoT5005C	1.28%
MitoG5047A	3.50%
MitoT5443C	1.01%
MitoT5496C	0.98%
MitoA5657G	1.27%
MitoG5774A	1.83%
MitoA5952G	1.76%
MitoG6027A	0.70%
MitoC6046T	1.01%
MitoG6261A	1.39%
MitoT6681C	1.17%
MitoT6777C	3.27%
MitoA7056G	3.00%
MitoT7176C	3.38%
MitoG7522A	9.87%
MitoG8617T	0.59%
MitoA8870G	0.24%
MitoA9073G	1.72%
MitoA9094G	0.41%
MitoG9378A	0.64%
MitoC9541T	20.61%
MitoA9668G	0.91%
MitoT9699C	6.39%
MitoT10035C	2.04%
MitoA10045G	0.65%
MitoG10311A	0.38%
MitoT10322C	1.22%
MitoG10399A	34.32%
MitoA10551G	6.12%
MitoG10590A	1.61%

MitoG10689A	4.29%
MitoC10874T	20.68%
MitoA11252G	15.00%
MitoG11378A	1.45%
MitoA11468G	16.93%
MitoT11900C	0.56%
MitoG11915A	8.80%
MitoA12309G	17.30%
MitoG12373A	17.43%
MitoG12631A	0.38%
MitoC12670T	0.08%
MitoT12706C	29.63%
MitoA13106G	8.08%
MitoA13264G	2.55%
MitoA13781G	2.07%
MitoT13790C	3.36%
MitoT14179C	3.56%
MitoA14234G	5.85%
MitoG15044A	6.73%
MitoC15536T	2.36%
MitoT15671C	1.07%
MitoA15759G	1.03%
MitoT15785C	4.05%
MitoA15925G	4.51%
MitoG15929A	7.66%
MitoG15931A	1.00%
MitoG16130A	9.15%
MitoT16145C	0.14%
MitoG16146A	3.35%
MitoC16149T	1.61%
MitoA16163G	1.73%
MitoA16164G	2.04%
MitoC16272T	8.38%

MitoC16329T	3.22%
MitoG16393A	2.12%
